# Effects of traditional Chinese medicine nursing techniques combined with music-based interventions on sleep in older adults: a systematic review and meta-analysis

**DOI:** 10.3389/fpsyt.2026.1836271

**Published:** 2026-06-11

**Authors:** Yupeng He, Yingcong Liu, Junlin He, Shan Zhu, Zhenhua Guo

**Affiliations:** 1 School of Music Education, Wuhan Conservatory of Music, Wuhan, Hubei, China; 2 Department of Music, Hansei University, Gunpo-si, Gyeonggi-do, Republic of Korea; 3 School of Pharmaceutical Sciences, Wenzhou Medical University, Wenzhou, Zhejiang, China; 4 Department of Design, Dong-A University, Busan, Republic of Korea

**Keywords:** meta-analysis, music-based interventions, older adults, sleep quality, systematic review, traditional Chinese medicine nursing techniques

## Abstract

**Objective:**

The aims of this study were to systematically evaluate the effects of traditional Chinese medicine nursing techniques combined with music-based interventions on sleep quality in older adults and to explore their effects on anxiety and pain.

**Methods:**

Randomized controlled trials (RCTs) published up to 9 May 2026 were systematically searched in China National Knowledge Infrastructure, Wanfang Data, VIP Database, China Biomedical Literature Database, PubMed, Web of Science, the Cochrane Library, and Embase. Meta-analysis was performed using RevMan 5.4 and Stata 15.0. The primary outcome was the Pittsburgh Sleep Quality Index (PSQI), and the secondary outcomes were the Self-Rating Anxiety Scale (SAS) and the Visual Analogue Scale (VAS). Fixed- or random-effects models were selected according to the degree of heterogeneity, and subgroup analyses were performed.

**Results:**

A total of 11 RCTs involving 838 older adults were included. The pooled results showed that the combined intervention significantly improved sleep quality [PSQI: mean difference (MD) = −2.28, 95% confidence interval (CI): −3.07 to −1.48]. Compared with the control group, the combined intervention was also associated with greater reductions in anxiety (SAS: MD = −9.95, 95% CI: −14.31 to −5.59) and pain (VAS: MD = −1.06, 95% CI: −1.57 to −0.54). Subgroup analyses showed that auricular acupressure, Baduanjin, and traditional Chinese medicine massage combined with music-based interventions all improved PSQI scores. Among these, auricular acupressure combined with music-based interventions showed the most stable effect (MD = −1.89, 95% CI: −2.55 to −1.23). Interventions lasting 15 days or longer were associated with lower heterogeneity and more stable effects.

**Conclusion:**

Traditional Chinese medicine nursing techniques combined with music-based interventions may improve sleep quality in older adults and may also help reduce anxiety and pain. Different combined approaches showed favorable effects, with auricular acupressure combined with music-based interventions demonstrating the most stable results. Interventions lasting 15 days or longer may be more likely to produce stable benefits.

**Systematic Review Registration:**

https://www.crd.york.ac.uk/prospero/, identifier CRD420261308801.

## 1 Introduction

With ongoing population aging, sleep health has become an important determinant of functional status and quality of life in older adults (1). Age-related changes in sleep architecture and circadian rhythms are common and are often accompanied by difficulty falling asleep, frequent nocturnal awakenings, early morning awakening, and reduced sleep efficiency (2). Poor sleep quality not only impairs daytime functioning and social participation (3) but also may be associated with mood disorders (4), cognitive decline (5), and poorer chronic disease management (6). Epidemiological studies have shown that approximately 20%–30% of community-dwelling older adults experience clinically significant insomnia or other sleep disorders (7), and that the prevalence may exceed 40% among residents of long-term care facilities (8).

Current interventions for sleep disorders in older adults mainly include pharmacological and non-pharmacological approaches (9). Pharmacological treatments primarily include benzodiazepines and related agents, melatonin and melatonin receptor agonists, and certain antidepressants. Although these medications may improve difficulties in sleep initiation and nocturnal awakenings in the short term, they may also cause adverse effects in older adults, including cognitive impairment, daytime drowsiness, an increased risk of falls, and drug dependence (10). In addition, because older adults often have multiple chronic conditions and are frequently exposed to polypharmacy, the potential for drug interactions should not be overlooked (11). Therefore, identifying safe and effective sleep interventions suitable for long-term use has become an important priority in geriatric health management and nursing practice.

In recent years, traditional Chinese medicine nursing techniques combined with music-based interventions have received increasing attention in the management of sleep problems in older adults (12). Previous studies have suggested that, compared with single-component interventions, this combined approach may offer greater benefits for improving subjective sleep quality, shortening sleep latency, and reducing nocturnal awakenings (13). These benefits may also be accompanied by reductions in anxiety, depression, and other negative emotional states (14), suggesting broader multidimensional effects. As a complementary approach to pharmacological treatment (15), traditional Chinese medicine nursing techniques include syndrome-differentiation-based nursing and the integrated use of methods such as acupoint stimulation, auricular acupressure, and foot bath therapy to regulate qi and blood, calm the mind, and improve physical comfort and emotional wellbeing, thereby promoting sleep (16). Music-based interventions, as non-invasive and low-cost adjunctive therapies, may regulate emotional arousal and autonomic nervous system activity (17), reduce subjective tension, relieve anxiety (18) and depression (19), and alleviate pain to some extent (20). Proposed mechanisms include reductions in cortisol levels, promotion of melatonin secretion, and enhancement of parasympathetic activity, which may shorten sleep latency and reduce nocturnal awakenings through multiple pathways (9, 21). The combined use of these two approaches may therefore provide a safer and more individualized model of sleep care for older adults.

However, existing combined intervention protocols vary substantially in their components, including the types of traditional Chinese medicine nursing techniques used, music selection, intervention frequency, and treatment duration. To date, no systematic review or meta-analysis has specifically examined the effects of traditional Chinese medicine nursing techniques combined with music-based interventions on sleep in older adults. Therefore, the overall efficacy of this combined approach and the strength of the available evidence remain unclear. Accordingly, this study conducted a systematic review and meta-analysis of randomized controlled trials (RCTs), with sleep quality as the primary outcome and anxiety and pain as secondary outcomes, to comprehensively evaluate the effects of traditional Chinese medicine nursing techniques combined with music-based interventions on sleep quality and related symptoms in older adults. In addition, subgroup analyses were performed to further explore the potential influence of intervention type and treatment duration on efficacy, with the aim of providing evidence to inform the clinical application of this combined approach in older adults.

## 2 Materials and methods

### 2.1 Search strategy

A systematic literature search was conducted in the following databases: China National Knowledge Infrastructure (CNKI), Wanfang Data, VIP Database (Chinese Scientific Journals Database), China Biomedical Literature Database (CBM), PubMed, Web of Science, the Cochrane Library, and Embase, from database inception to 9 May 2026. The Chinese search terms included “音乐治疗” (music therapy), “音乐干预” (music intervention), “音乐疗法” (music therapy), “五音疗法” (five-tone therapy), “睡眠障碍” (sleep disorders), “失眠” (insomnia), “不寐” (insomnia), “睡眠质量” (sleep quality), “老年” (older adults), “老年人” (older adults), “老年疾病” (geriatric diseases), and “养老” (elderly care). The English search terms included “Music”, “Music intervention”, “Music therapy”, “Sleep quality”, “Sleep–wake disorders”, “Short sleep phenotypes”, “Aged”, and “Elderly”. Subject headings and free-text terms were combined in the search. The full search strategy is provided in the Supplementary Materials.

### 2.2 Inclusion and exclusion criteria

Studies were included if they met the following criteria:

1. Participants: Older adults aged ≥60 years with sleep problems, including insomnia symptoms, decreased sleep quality, or other sleep-related complaints reported in the original studies. Because the included studies did not uniformly report formal diagnostic criteria for sleep disorders, no further restrictions were placed on diagnostic status.
2. Interventions: The experimental group received traditional Chinese medicine nursing techniques combined with music-based interventions. Traditional Chinese medicine nursing techniques mainly referred to non-pharmacological traditional Chinese medicine approaches delivered by nursing staff, including auricular acupressure, Baduanjin, traditional Chinese medicine massage, Chinese herbal foot bath, and traditional Chinese medicine emotional nursing, whereas acupuncture and oral Chinese herbal medicine were excluded.
3. Controls: The control group received routine care or conventional interventions, including routine nursing care (psychological counseling, health education, and lifestyle guidance), sleep health education (sleep hygiene education and sleep guidance), and pharmacological treatment.
4. Study design: RCTs.
5. Language: Studies published in Chinese or English.

Studies were excluded if they met any of the following criteria:

1. Duplicate publications or studies based on overlapping data.
2. Protocols, reviews, including systematic reviews, theoretical articles, case reports, or conference abstracts.
3. Studies in which music-based interventions were used alone rather than in combination with traditional Chinese medicine nursing techniques.

The primary outcome was the Pittsburgh Sleep Quality Index (PSQI). The secondary outcomes were the Self-Rating Anxiety Scale (SAS) and the Visual Analogue Scale (VAS).

### 2.3 Data extraction

All retrieved records were imported into EndNote 21 for deduplication. Titles and abstracts were screened according to the predefined inclusion and exclusion criteria, and clearly ineligible studies were excluded. The full texts of potentially eligible studies were then reviewed to determine final inclusion. Three researchers independently extracted the following data: author, year of publication, sample size, participant characteristics (age and sex), intervention and control measures, intervention intensity (frequency, duration, and treatment course), and outcome data. Any disagreements were resolved through discussion and, when necessary, consultation with a fourth researcher.

### 2.4 Risk of bias assessment

The methodological quality of the included studies was independently assessed by three researchers using the Cochrane Risk of Bias tool. The following domains were evaluated: random sequence generation, allocation concealment, blinding, completeness of outcome data, selective reporting, and other sources of bias. Each domain was judged as having a low, high, or unclear risk of bias. Any disagreements were resolved through discussion.

### 2.5 Data synthesis and statistical analysis

Meta-analysis was conducted using RevMan 5.4 and Stata 15.0. Continuous outcomes were expressed as mean differences (MDs) with 95% confidence intervals (CIs) and pooled using the inverse-variance method. Heterogeneity was assessed using the χ² test and the *I*² statistic. When *I*² > 50% or *p* < 0.10, a random-effects model was applied, and τ² was estimated using the DerSimonian–Laird method; otherwise, a fixed-effects model was used. When at least 10 studies were included, funnel plots were generated and Egger’s regression test was performed to assess potential publication bias. When the number of included studies was limited, especially when fewer than four studies were included in an outcome or subgroup analysis, the stability and generalizability of the pooled estimates may be limited, and the findings were mainly interpreted as exploratory and with caution.

## 3 Results

### 3.1 International prospective register of systematic reviews registration

This systematic review and meta-analysis was registered in the International Prospective Register of Systematic Reviews (PROSPERO; registration number: CRD420261308801). The study design, implementation, and reporting followed the PRISMA 2020 statement, and the processes of study selection, risk of bias assessment, and data synthesis were conducted with reference to Cochrane methodological guidance for systematic reviews.

### 3.2 Study selection

**Figure 1:**
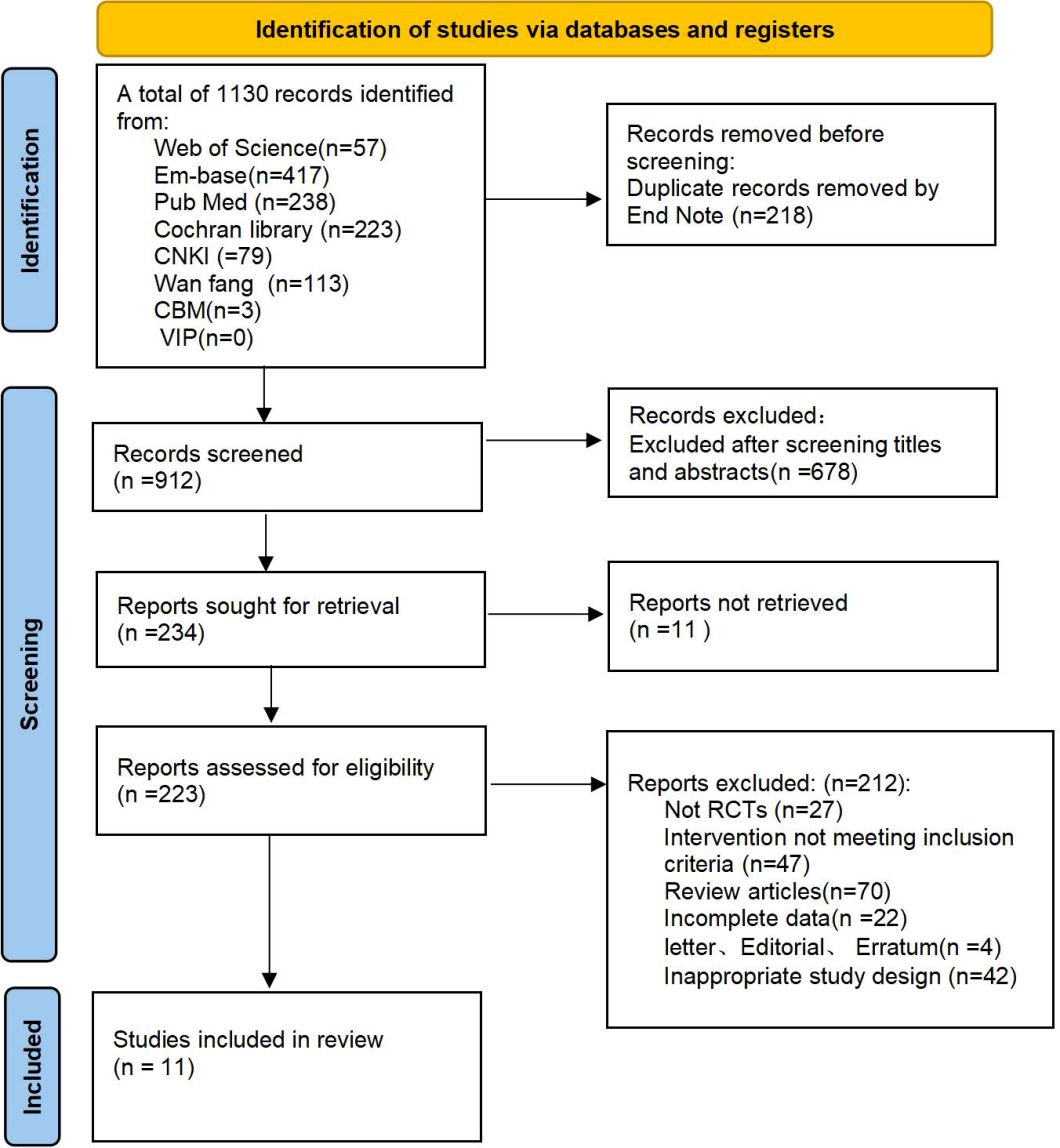
PRISMA 2020 flow diagram of the study selection process.

After screening, 11 studies published in Chinese and English were included, involving a total of 838 participants, with 419 participants in the intervention group and 419 in the control group. The study selection process is shown in **Figure 1**, and the basic characteristics of the included studies are presented in **Table 1**.

**Table 1:** Characteristics of the included studies.

Source	Group size (age)		Sex		Intervention description		Duration of combined treatment (days)	Frequency of TCM nursing technique	Duration per TCM nursing session (min)	Frequency of music intervention (per week)	Duration of music intervention (min)	Outcomes
	IG	CG	IG: male/ female	CG: male/ female	IG	CG						
Miao Xiaohong 2018 (22)	30 (73.1 ± 5.77)	30 (75.23 ± 4.85)	23/7	20/10	Baduanjin + music listening *	TAU	28	5/week	30	5	30	PSQI
Zhou Sumei 2019 (23)	36 (65.2 ± 8.28)	36 (67.60 ± 8.23)	14/22	13/23	Auricular acupressure + music listening *	Auricular acupressure	28	3/day	3–5	7	45	PSQI
Liao Tao 2019 (24)	30 (65-70)	30 (65-70)	NR	NR	Auricular acupressure + music listening *	TAU	30	6/day	Approximately 20 s per acupoint	5	30	PSQI, SAS, VAS
Liu Yongli 2019 (25)	39 (66.82 ± 3.6)	39 (66.82 ± 3.60)	18/21	20/19	Chinese herbal foot bath + music listening ^#^	SHE	30	7/week	30	7	30	PSQI
Zhan Wei 2020 (26)	69 (70.6 ± 4.5)	69 (70.2 ± 4.1)	32/37	30/39	Auricular acupressure + music listening ^&^+ TAU	TAU	30	5/day	1	7	30	PSQI
Wang Xin 2021 (27)	30 (68.3 ± 7. 9)	30 (69. 8 ± 9. 4)	17/13	19/11	Auricular acupressure + music listening ^#^	Pharmacological treatment + SHE	56	4–6/day	3–5	14	60	PSQI
Wang Feifeng 2022 (28)	30 (69.43 ± 3.46)	30 (70.35 ± 2.84)	19/11	17/13	TCM massage + music listening ^#^	TAU	NR	NR	3	5	20	PSQI
Wei Xiaoying 2023 (29)	52 (73.12 ± 3.49)	52 (73.26 ± 3.55)	28/24	27/25	TCM massage + music listening^&^	TAU	7	2/day	5	7	30	PSQI,SAS, VAS
Cheng Yun 2024 (30)	30(76.5 ± 2.1)	30(73 ± 2.6)	14/16	12/18	TCM emotional nursing + music listening ^#^	TAU	9	Once every 3 days	15–20	7	15–30	PSQI, SAS
Zhu Shu 2024 (31)	25 (67.08 ± 11.58)	25 (68.24 ± 6.84)	14/11	9/16	Auricular acupressure + music listening ^&^	TAU	7	3–5/day	2	NR	60	PSQI
Pan Xuehong 2025 (32)	48 (71.04 ± 4.51)	48 (70.69 ± 4.2)	26/22	25/23	Baduanjin + GIM*	TAU	28	5/week	30	NR	20–30	PSQI

IG, intervention group; CG, control group; TCM, traditional Chinese medicine; TAU, treatment as usual; SHE, sleep health education; GIM, Guided Imagery and Music; NR, not reported; PSQI, Pittsburgh Sleep Quality Index; SAS, Self-Rating Anxiety Scale; VAS, Visual Analogue Scale.

Music selection methods: *self-selected; ^#^therapist-selected; ^&^not specified.

### 3.3 Risk of bias assessment

**Figure 2:**
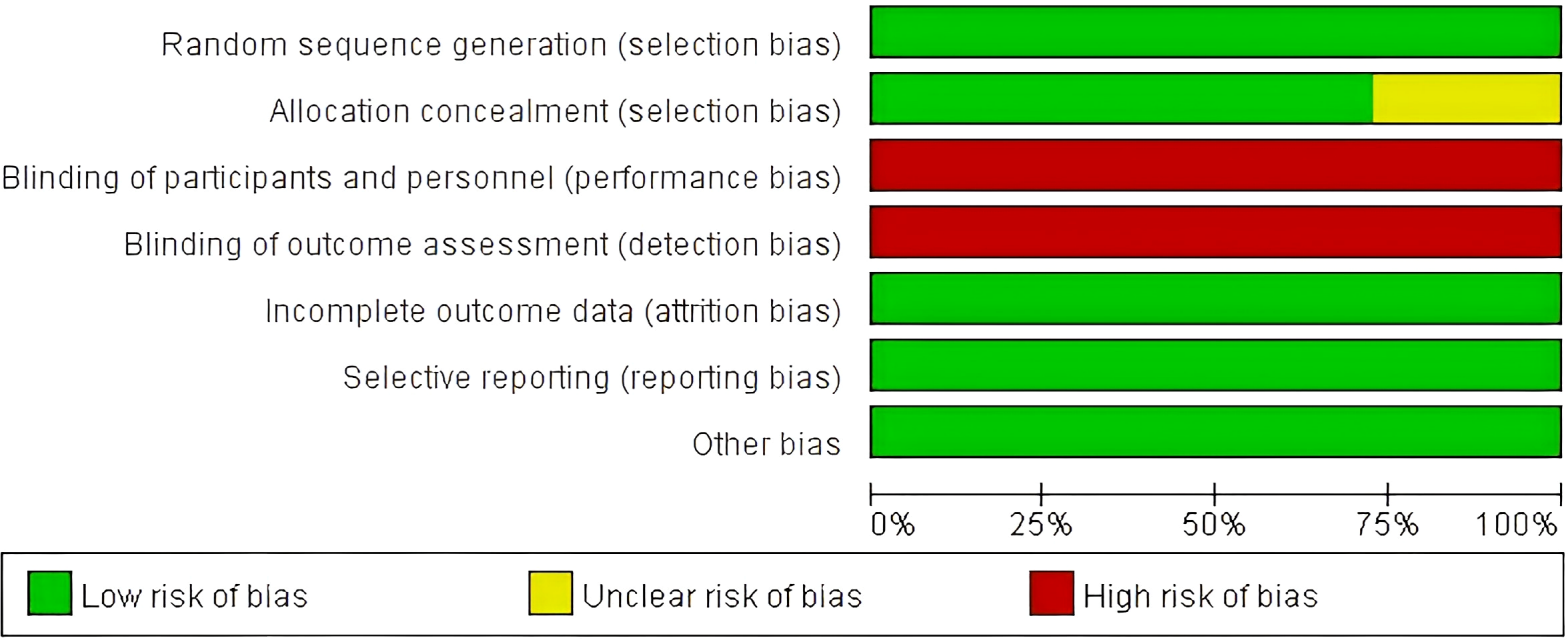
Risk of bias graph.

**Figure 3:**
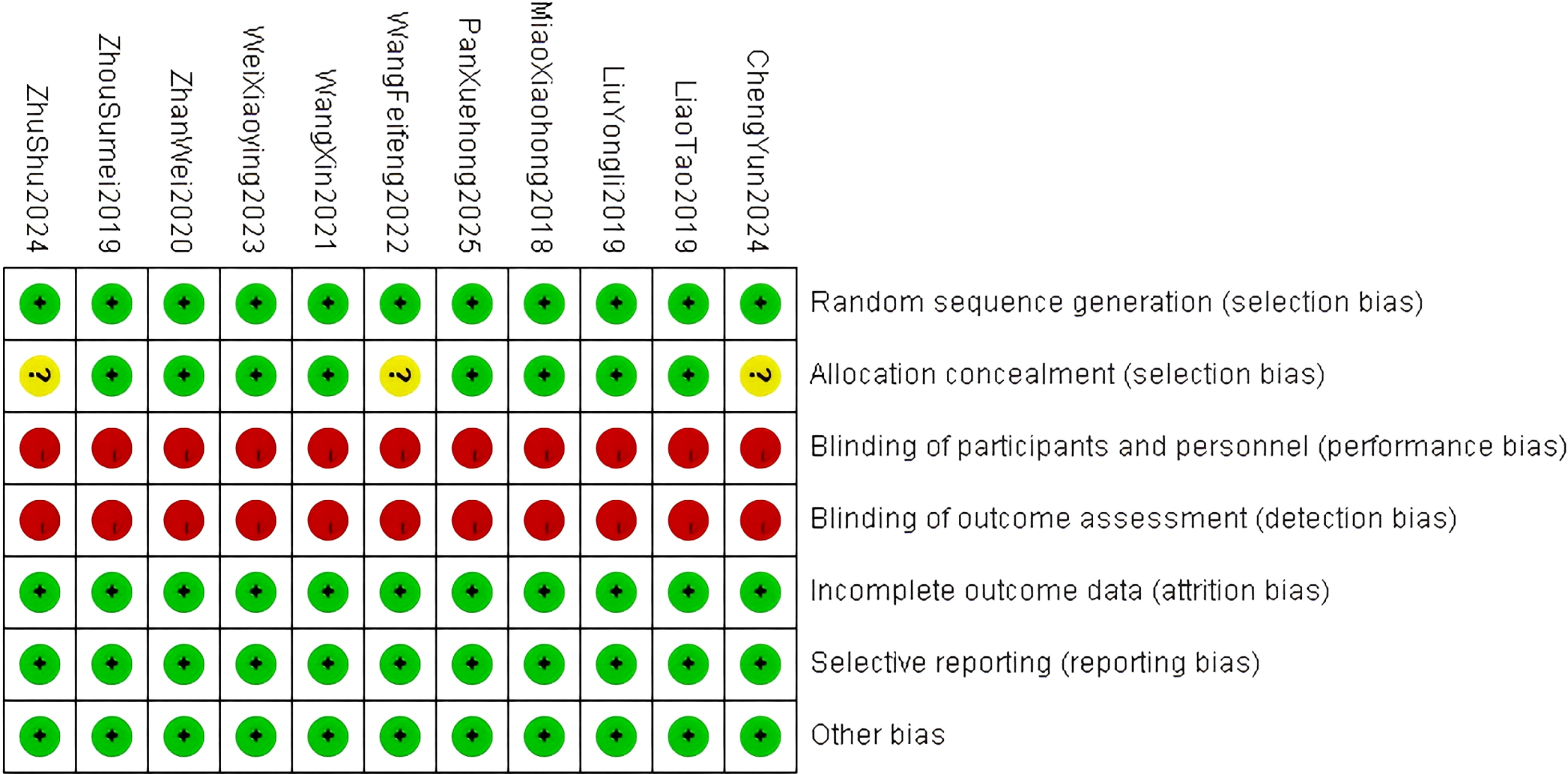
Risk of bias summary.

The methodological quality of the included studies was assessed using the Cochrane Risk of Bias tool. Among the 11 included studies (22–32) (**Figure 2** and **3**), 8 studies (22–27, 29, 32) reported appropriate methods of random sequence generation, including random number tables, computer-generated randomization, or envelope-based allocation. None of the studies clearly reported blinding of participants, intervention personnel, or outcome assessors. Although blinding of participants may be difficult to implement in interventions involving music combined with Baduanjin or traditional Chinese medicine massage, blinding of outcome assessors is feasible; the absence of such reporting may increase the risk of measurement bias. Overall, outcome data were generally complete, and no other obvious sources of bias were identified.

### 3.4 Meta-analysis results

#### 3.4.1 Effects of traditional Chinese medicine nursing techniques combined with music-based interventions on sleep quality

**Figure 4:**
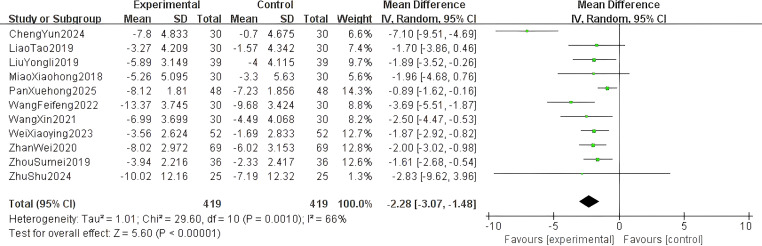
Forest plot of the effects of traditional Chinese medicine nursing techniques combined with music-based interventions on sleep quality (PSQI).

A total of 11 studies (22–32) evaluated the effects of traditional Chinese medicine nursing techniques combined with music-based interventions on sleep quality in older adults. The pooled analysis showed substantial heterogeneity across studies (*I*² = 66%, *p* = 0.001); therefore, a random-effects model was used. As shown in **Figure 4**, the combined intervention group showed greater improvement in sleep quality than the control group (MD = −2.28, 95% CI: −3.07 to −1.48), and the overall effect was statistically significant (*p* < 0.00001).

**Figure 5:**
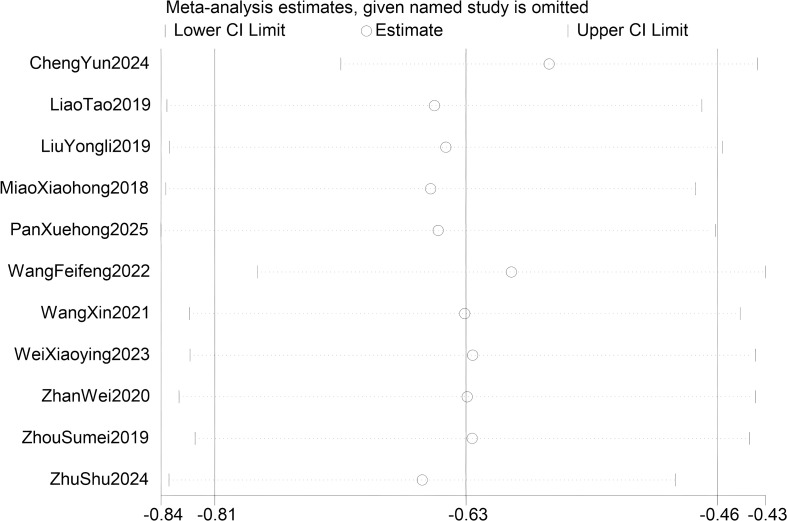
Sensitivity analysis of the effects of traditional Chinese medicine nursing techniques combined with music-based interventions on sleep quality (PSQI).

**Figure 6:**
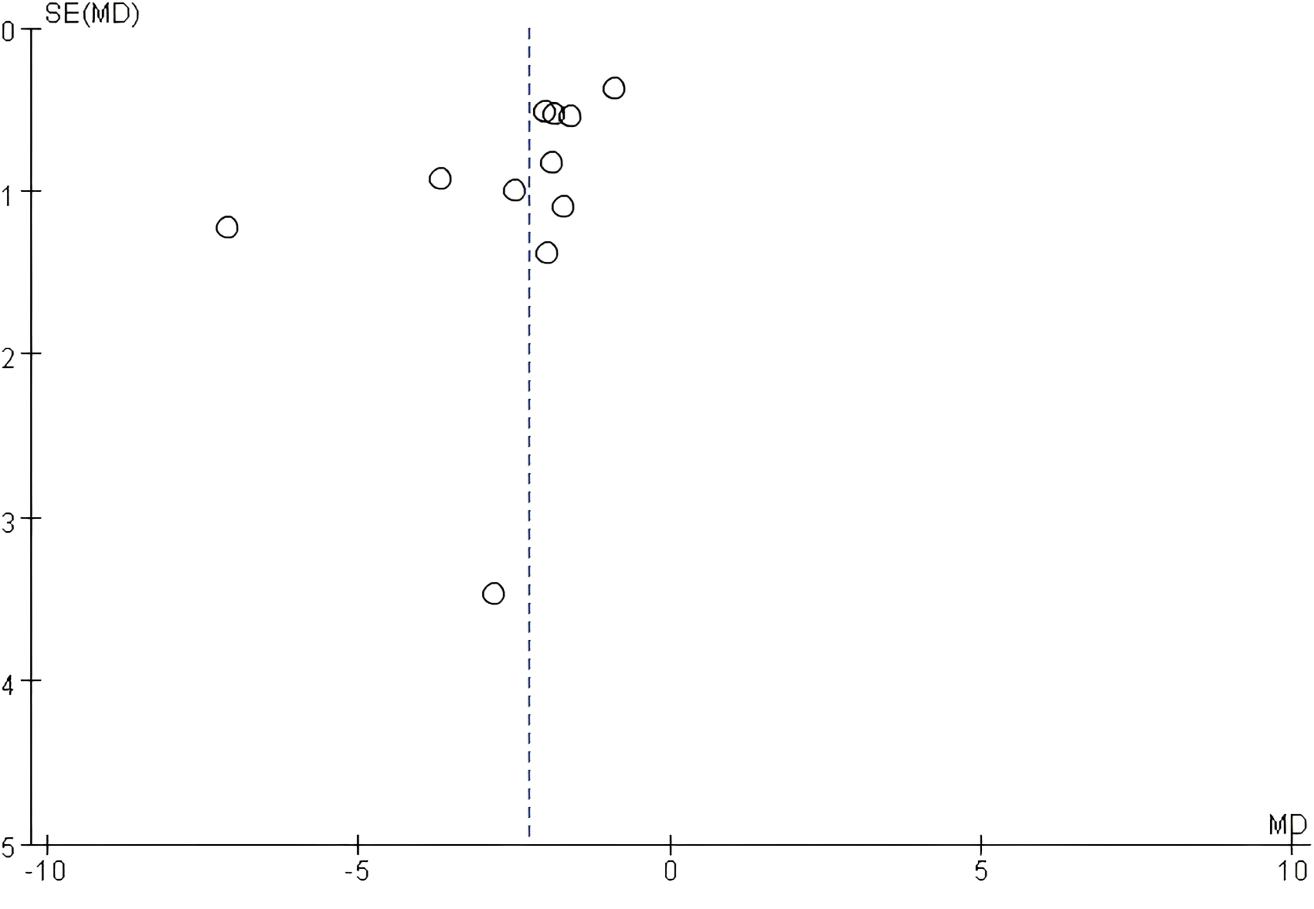
Funnel plot of the effects of traditional Chinese medicine nursing techniques combined with music-based interventions on sleep quality (PSQI).

Leave-one-out sensitivity analysis showed that the pooled effect estimates remained statistically significant and consistent in direction after the sequential omission of individual studies, indicating that the findings had a certain degree of stability (**Figure 5**). Among the included studies, Cheng Yun (2024) showed a larger effect size than the other studies. After excluding this study and repeating the sensitivity analysis, the pooled effect remained negative, and the effect estimates changed only slightly after the sequential omission of the remaining studies. This suggests that the overall result was not dominated by this single study, although it may have contributed to the heterogeneity of the PSQI outcome. Visual inspection of the funnel plot did not reveal obvious asymmetry (**Figure 6**). Egger’s regression test showed an intercept of −1.32 (95% CI: −6.69 to 4.05), with *t* = −0.56 and *p* = 0.591, suggesting that there were no statistically significant small-study effects.

#### 3.4.2 Effects of traditional Chinese medicine nursing techniques combined with music-based interventions on anxiety

**Figure 7:**
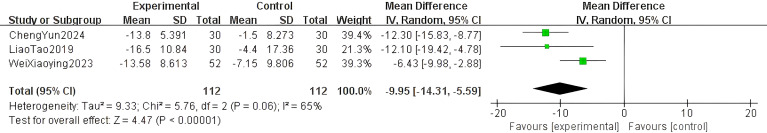
Forest plot of the effects of traditional Chinese medicine nursing techniques combined with music-based interventions on anxiety (SAS).

Three studies (24, 29, 30) reported the effects of the combined intervention on anxiety in older adults. Substantial heterogeneity was observed across studies (*I*² = 65%, *p* = 0.06); therefore, a random-effects model was used. As shown in **Figure 7**, the combined intervention group showed a greater reduction in SAS scores than the control group (MD = −9.95, 95% CI: −14.31 to −5.59), and the overall effect was statistically significant (*p* < 0.00001).

#### 3.4.3 Effects of traditional Chinese medicine nursing techniques combined with music-based interventions on pain

**Figure 8:**
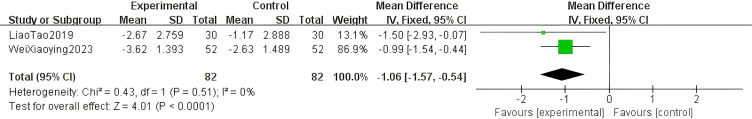
Forest plot of the effects of traditional Chinese medicine nursing techniques combined with music-based interventions on pain (VAS).

Two studies (24, 29) reported the effects of the combined intervention on pain in older adults. No heterogeneity was observed across studies (*I*² = 0%, *p* = 0.51); therefore, a fixed-effects model was used. As shown in **Figure 8**, the combined intervention group showed a greater reduction in VAS scores than the control group (MD = −1.06, 95% CI: −1.57 to −0.54), and the overall effect was statistically significant (*p* < 0.0001).

### 3.5 Subgroup analysis

To explore the effects of different types of combined interventions on total PSQI scores and heterogeneity, subgroup analyses were conducted according to intervention type, including auricular acupressure combined with music-based interventions, Baduanjin combined with music-based interventions, and traditional Chinese medicine massage combined with music-based interventions (**Table 2**).

**Table 2:** Subgroup analyses of the effects of traditional Chinese medicine nursing techniques combined with music-based interventions on sleep quality (PSQI).

Subgroups	No. of studies	Total sample (I+C)	Heterogeneity		MD (95% CI)	Meta-analysis results	
			*I*^2^ (%)	*p*-value		*Z*	*p*
Type of intervention							
Auricular acupressure +music-based interventions	5	380	0	0.94	-1.89 [-2.55, -1.23]	5.64	<0.00001
Baduanjin + music-based interventions	2	156	0	0.46	-0.96 [-1.67, -0.25]	2.66	0.008
TCM massage + music-based interventions	2	164	65	0.09	−2.62 [−4.38, −0.87]	2.93	0.003
Duration of intervention (days)							
<15	3	214	87	0.0005	−4.04 [−8.22, 0.14]	1.89	0.06
15–29	3	228	0	0.46	−1.16 [−1.75, −0.57]	3.85	0.0001
≥30	4	336	0	0.95	−2.01 [−2.76, −1.27]	5.3	<0.00001

IG, intervention group; CG, control group; TCM, traditional Chinese Medicine; PSQI, Pittsburgh Sleep Quality Index; MD, mean difference; CI, confidence interval.

The results showed that auricular acupressure combined with music-based interventions (five studies, *n* = 380) significantly improved total PSQI scores compared with the control group (MD = −1.89, 95% CI: −2.55 to −1.23, *p* < 0.00001), with no observed heterogeneity (*I*² = 0%, *p* = 0.94). Baduanjin combined with music-based interventions (two studies, *n* = 156) also showed a significant effect (MD = −0.96, 95% CI: −1.67 to −0.25, *p* = 0.008), with no observed heterogeneity (*I*² = 0%, *p* = 0.46). Traditional Chinese medicine massage combined with music-based interventions (two studies, *n* = 164) also improved total PSQI scores (MD = −2.62, 95% CI: −4.38 to −0.87, *p* = 0.003), although heterogeneity was relatively high (*I*² = 65%, *p* = 0.09). Overall, different types of combined interventions were associated with improved sleep quality in older adults, with auricular acupressure combined with music-based interventions showing the most stable results.

Considering the differences in the number of studies across intervention approaches, subgroup analysis was performed according to treatment duration (<15 days, 15–29 days, and ≥30 days) (**Table 2**). The results showed that, in the <15-day subgroup (three studies, *n* = 214), the difference in PSQI scores did not reach statistical significance (*p* = 0.06), and within-subgroup heterogeneity was substantial (*I*² = 87%, *p* = 0.0005). For interventions lasting 15–29 days (three studies, *n* = 228), a significant improvement was observed (MD = −1.16, 95% CI: −1.75 to −0.57, *p* = 0.0001), with no observed heterogeneity (*I*² = 0%, *p* = 0.46). For interventions lasting ≥30 days (four studies, *n* = 336), significant effects were also observed (MD = −2.01, 95% CI: −2.76 to −1.27, *p* < 0.00001), with no observed heterogeneity (*I*² = 0%, *p* = 0.95). Overall, studies with medium- to long-term interventions (≥15 days) showed more stable results, whereas the short-term intervention subgroup (<15 days) did not show a statistically significant difference and had substantial heterogeneity. Therefore, the subgroup findings should be interpreted with caution.

## 4 Discussion

This study systematically evaluated the effects of traditional Chinese medicine nursing techniques combined with music-based interventions on sleep quality in older adults. A total of 11 RCTs involving 838 participants were included. The meta-analysis showed that the combined intervention significantly improved PSQI scores and was associated with greater reductions in anxiety (SAS) and pain (VAS) than the control conditions, suggesting that this approach may provide multidimensional benefits.

### 4.1 Effects of traditional Chinese medicine nursing techniques combined with music-based interventions on sleep quality in older adults and influencing factors

Previous studies have shown that auricular acupressure may regulate autonomic nervous system activity, reduce nocturnal hyperarousal, and thereby improve sleep continuity (33). RCTs using polysomnography have reported that auricular acupressure can increase slow-wave sleep and reduce physiological stress (34). In older adults with chronic diseases, this intervention has also shown potential benefits for sleep outcomes (35). Baduanjin, as a gentle mind–body exercise, may improve autonomic balance by enhancing heart rate variability through moderate physical activity and rhythmic breathing and movement, promoting melatonin secretion, and shortening sleep latency (36, 37). A systematic review and meta-analysis in older adults with insomnia further suggested that Baduanjin may improve both total PSQI scores and dimensional scores and that longer intervention duration may be associated with more stable effects (38). A systematic review by Khaleghi and Ahmadi also showed that various forms of exercise, including Tai Chi, Qigong, and Baduanjin, may help improve sleep quality in older women, further supporting the potential value of exercise-based traditional Chinese medicine nursing techniques in sleep interventions for older adults (39). Traditional Chinese medicine massage may reduce muscle tension and discomfort through tactile stimulation, induce a relaxation response, decrease sympathetic nervous system activity, and prolong sleep duration (40, 41). In addition, music-based interventions may reduce pre-sleep hyperarousal, promote relaxation, and improve sleep structure, including subjective sleep quality and related sleep dimensions (42, 43).

Consistent with these mechanisms, the present meta-analysis showed that traditional Chinese medicine nursing techniques combined with music-based interventions significantly reduced PSQI scores compared with routine control measures, indicating a potential advantage in improving subjective sleep quality in older adults. Traditional Chinese medicine nursing techniques primarily target physiological and somatic arousal, whereas music-based interventions mainly reduce pre-sleep cognitive and emotional tension and hyperarousal (44). These complementary mechanisms may allow the combined approach to address multiple factors involved in the maintenance of sleep disturbances, thereby providing broader benefits than single-component interventions. Accordingly, subgroup analyses stratified by intervention type and duration were conducted to examine the influence of different technique combinations and intervention intensity on effect size and result stability and to provide a basis for more targeted clinical application.

Subgroup analysis by intervention type showed that auricular acupressure combined with music-based interventions had particularly pronounced effects on total PSQI scores (**Table 2**), with no observed heterogeneity (*I*² = 0%), indicating good stability. This advantage may be related to modulation of central autonomic regulatory systems through auricular afferent pathways and associated brainstem structures (45). In addition, auricular acupressure involves relatively standardized acupoint selection, stimulation procedures, and operational processes, which may improve reproducibility in clinical practice (46). By contrast, exercise-based interventions such as Baduanjin may be influenced by individual differences in movement amplitude, rhythm control, and adherence, whereas traditional Chinese medicine massage may vary according to practitioner skill and force application (47).

Further subgroup analysis according to intervention duration indicated that combined interventions lasting ≥15 days produced more stable improvements in total PSQI scores. Improvements in sleep quality often involve gradual behavioral adjustment and the reconstruction of subjective sleep perception. Therapeutic effects may accumulate progressively over the course of the intervention, whereas excessively short intervention durations may be insufficient to achieve stable and sustained improvements (48, 49). This finding is generally consistent with previous meta-analyses (38, 50).

### 4.2 Effects of traditional Chinese medicine nursing techniques combined with music-based interventions on anxiety and influencing factors

Neuroimaging evidence indicates that music can activate the prefrontal cortex, limbic system, and reward-related networks, thereby influencing the intensity of emotional responses and emotional evaluation processes (51, 52). Systematic reviews by Salihu and Wang have also reported that music-based interventions may significantly reduce anxiety and improve emotional states by decreasing subjective tension and physiological stress responses (53, 54). Meanwhile, traditional Chinese medicine nursing techniques may indirectly regulate emotional states by reducing physiological tension and somatic burden. Specifically, mind–body exercises such as Baduanjin may enhance self-efficacy and physical comfort, thereby reducing anxiety and depression scores in older adults (55). Tactile interventions such as auricular acupressure and traditional Chinese medicine massage may induce relaxation and reduce physiological tension, thereby contributing to emotional improvement (56). The meta-analysis showed that traditional Chinese medicine nursing techniques combined with music-based interventions significantly reduced anxiety (SAS) scores in older adults, suggesting potential benefits for anxiety regulation. Compared with single-component interventions, the combined approach may offer complementary pathways by simultaneously targeting emotional processes and arousal-related mechanisms, thereby facilitating improvement in the complex psychosomatic conditions commonly observed in older adults.

### 4.3 Effects of traditional Chinese medicine nursing techniques combined with music-based interventions on pain and influencing factors

Music stimulation can modulate activity in pain-related brain regions, including the prefrontal cortex, anterior cingulate cortex, and insula, thereby influencing both pain intensity and the affective dimension of pain (57). Further evidence suggests that music may enhance endogenous analgesia through activation of the endogenous opioid system and dopaminergic reward pathways (58). Among the VAS outcomes included in this study, the pain-related traditional Chinese medicine nursing techniques mainly involved auricular acupressure and traditional Chinese medicine massage. In auricular interventions, sensory afferents in the auricle are connected to vagus nerve-related pathways and may participate in the modulation of central pain regulatory networks. Recent systematic reviews and meta-analyses of auricular vagus nerve stimulation (aVNS) and transcutaneous auricular vagus nerve stimulation (taVNS) have indicated potential analgesic effects across a range of chronic pain conditions (59, 60). Regarding traditional Chinese medicine massage, mechanoreceptor input from superficial and deep tissues may influence pain-gating mechanisms and central pain processing (61, 62). In addition, as a tactile intervention capable of inducing a relaxation response, traditional Chinese medicine massage may also reduce tension-related physiological burden, thereby alleviating the emotional–somatic interaction component of pain (56, 63).

Based on these mechanisms, the meta-analysis indicated that traditional Chinese medicine nursing techniques combined with music-based interventions reduced VAS scores in older adults. Because pain includes both sensory-discriminative and affective components, the combined modulation produced by music-based interventions and auricular or manual techniques may contribute to broader analgesic effects.

However, the number of included studies on pain was limited, and differences were observed in pain types and intervention forms. Therefore, the pooled effect should be interpreted with caution. Future studies should further distinguish among pain types, standardize intervention protocols, and incorporate objective physiological indicators to clarify the independent contribution of this combined intervention to pain outcomes.

Finally, safety remains an important issue when interpreting the findings of this study. Although traditional Chinese medicine nursing techniques combined with music-based interventions are non-pharmacological interventions and are generally considered to have a relatively low overall risk, this does not mean that they are completely free of adverse events. Previous studies have shown that exercise-based interventions such as Baduanjin may be associated with discomforts such as palpitations, dizziness, knee pain, low back pain, and fatigue (64). Adverse events related to auricular acupressure mainly include local skin irritation, pain, discomfort, mild tenderness, or dizziness (65). However, none of the studies included in this review systematically reported adverse events, withdrawals due to adverse events, or safety monitoring procedures. Therefore, the safety of the combined intervention could not be quantitatively evaluated. Future studies should standardize the recording and reporting of adverse events, reasons for withdrawal, and safety monitoring procedures while evaluating efficacy, so as to provide a more comprehensive assessment of both the effectiveness and safety of this combined intervention.

## 5 Conclusion

This systematic review and meta-analysis synthesized evidence from 11 RCTs. The findings suggest that traditional Chinese medicine nursing techniques combined with music-based interventions may improve sleep quality in older adults, as reflected by reduced PSQI scores, and may also have beneficial effects on anxiety (SAS) and pain (VAS).

Subgroup analyses indicated that different types of traditional Chinese medicine nursing techniques combined with music-based interventions were associated with improvements in sleep quality, with auricular acupressure combined with music-based interventions showing the most stable findings. In terms of intervention duration, medium- to long-term interventions (≥15 days) appeared to produce more stable effects, suggesting that sustained intervention may contribute to more durable improvements.

However, several limitations should be acknowledged. First, the number of studies included for secondary outcomes, such as anxiety and pain, and for some subgroup analyses was relatively small, which may affect the stability and generalizability of the findings; therefore, these results should be interpreted with caution. Second, none of the included studies clearly reported blinding of participants, intervention providers, or outcome assessors. This may increase the risk of measurement bias in outcomes assessed using subjective scales and may influence the estimated effect sizes. Third, the specific procedures of music-based interventions and traditional Chinese medicine nursing techniques were not standardized. In addition, none of the included studies systematically reported adverse events, withdrawals due to adverse events, or safety monitoring procedures. These limitations reduce the reproducibility and generalizability of the findings and also restrict the assessment of the safety of the combined intervention. Fourth, some studies lacked sex-stratified outcome data, and therefore, sex-based subgroup analyses could not be performed. Future studies should conduct more high-quality, large-sample RCTs to further verify the effects of the combined intervention on secondary outcomes, such as anxiety and pain, as well as across different subgroups. In addition, future research should standardize intervention protocols and reporting standards, provide sex-stratified outcome data, and extend follow-up periods to clarify the long-term effects and applicable populations of the combined intervention.

## Data Availability

The original contributions presented in the study are included in the article/**Supplementary Material**. Further inquiries can be directed to the corresponding author.
